# Kallikrein Transduced Mesenchymal Stem Cells Protect against Anti-GBM Disease and Lupus Nephritis by Ameliorating Inflammation and Oxidative Stress

**DOI:** 10.1371/journal.pone.0067790

**Published:** 2013-07-23

**Authors:** Yajuan Li, Indu Raman, Yong Du, Mei Yan, Soyoun Min, Jichen Yang, Xiangdong Fang, Wei Li, Jianxin Lu, Xin J. Zhou, Chandra Mohan, Quan-Zhen Li

**Affiliations:** 1 Department of Immunology and Internal Medicine, The University of Texas Southwestern Medical Center, Dallas, Texas, United States of America; 2 Quantitative Biomedical Research Center, The University of Texas Southwestern Medical Center, Dallas, Texas, United States of America; 3 Laboratory of Disease Genomics and Individualized Medicine, Beijing Institute of Genomics, Chinese Academy of Sciences, Beijing, China; 4 Key Laboratory of Medical Genetics, Wenzhou Medical College School of Laboratory Medicine & Life Science, Wenzhou, China; 5 Renal Path Diagnostics, Pathologist BioMedical Laboratories, Lewisville, Texas, United States of America; 6 BME Departments, University of Houston, Houston, Texas, United States of America; UAE University, Faculty of Medicine & Health Sciences, United Arab Emirates

## Abstract

Previously we have shown that kallikreins (klks) play a renoprotective role in nephrotoxic serum induced nephritis. In this study, we have used mesenchymal stem cells (MSCs) as vehicles to deliver klks into the injured kidneys and have measured their therapeutic effect on experimental antibody induced nephritis and lupus nephritis. Human KLK-1 (hKLK1) gene was transduced into murine MSCs using a retroviral vector to generate a stable cell line, hKLK1-MSC, expressing high levels of hKLK1. *129/svj* mice subjected to anti-GBM induced nephritis were transplanted with 10^6^ hKLK1-MSCs and hKLK1 expression was confirmed in the kidneys. Compared with vector-MSCs injected mice, the hKLK1-MSCs treated mice showed significantly reduced proteinuria, blood urea nitrogen (BUN) and ameliorated renal pathology. Using the same strategy, we treated lupus-prone *B6.Sle1.Sle3* bicongenic mice with hKLK1-MSCs and demonstrated that hKLK1-MSCs delivery also attenuated lupus nephritis. Mechanistically, hKLK1-MSCs reduced macrophage and T-lymphocyte infiltration into the kidney by suppressing the expression of inflammation cytokines. Moreover, hKLK1 transduced MSCs were more resistant to oxidative stress-induced apoptosis. These findings advance genetically modified MSCs as potential gene delivery tools for targeting therapeutic agents to the kidneys in order to modulate inflammation and oxidative stress in lupus nephritis.

## Introduction

Systemic lupus erythematosus (SLE) is a complex autoimmune disease which is characterized by autoantibody formation, immune complex deposition and chronic inflammatory processes involving multiple organs, especially the kidney. Lupus nephritis (LN) is a major clinical manifestation of SLE, occurring in more than 60% of patients [1]. The pathogenesis of LN involves a complex interaction of multiple factors, such as environmental triggers, hormonal factors, and susceptibility genes including genes that affect apoptosis, antigen/immune complex clearance, and inflammatory responses involving a variety of cytokines, chemokines and adhesion molecules [2]–[4]. Although suppression of systemic autoimmune responses is still the primary treatment strategy and immunosuppressive and anti-inflammatory agents have been used to induce disease remission in patients with lupus nephritis, some patients do not respond to these agents or suffer from generalized immunosuppression [5]. Hence, therapy for SLE and immune-mediated nephritis remains a challenge.

Recently, we have identified tissue kallikreins (klk) as lupus-susceptibility genes which are associated with experimental anti-GBM antibody-induced nephritis (EAN) and LN [6], [7]. Impaired up-regulation of klk or blocking the klk B2 receptor renders the mice sensitive to EAN, while infusion of bradykinin, the mediator of kallikrein signals, or systemic delivery of mouse klk1 gene using an adenoviral vector protected EAN-sensitive mice from developing nephritis. Moreover, we also noted that polymorphisms in human KLK-1 gene may be associated with human SLE [7]. These data suggested that kallikreins play a role in immune-mediated nephritis and could potentially be used as a therapeutic agent for the prevention and treatment of this disease. In order to develop this as a therapeutic strategy, we have tested various approaches including systemic delivery of klk1 using an adenoviral vector [6] or osmotic-pump-release of bradykinins [7]. Although the protective effect of klk against EAN is clear, delivering klk systemically for prolonged duration poses several problems including the poor stability of klk *in vivo* as well as the systemic side-effects of klk [8], [9]. In this study, we sought an alternative delivery strategy for prolonged biologic effect within inflamed end organs.

Stem cell therapy holds great promise for the repair of injured tissue and organs, including kidney [10]–[15]. It is reported that bone marrow (BM)-derived MSCs are renotrophic and have the capacity to differentiate into mesangial cells *in vivo* following glomerular injury [15], [16]. MSC transplantation has been shown to be effective in amelioration of renal damages caused by inflammatory and ischemic conditions [14], [15]. Moreover, MSCs genetically modified with the anti-apoptotic Akt gene or the anti-oxidant enzyme heme oxygenase (HO)-1 gene were more efficient in improving cardiac performance and healing damaged myocardium compared to unmodified MSCs by enhancing stem cell viability via autocrine and paracrine actions [17], [18]. Furthermore, kallikrein modified MSCs have been shown to have enhanced protection against acute ischemic kidney injury by inhibiting apoptosis and inflammation [19]. The above experimental results indicated that genetically modified MSCs can serve as ideal vehicle for delivering target genes to sites of tissue injury and provide advanced benefits in protection against ischemic injury. In the present study, we evaluated the protective effect of hKLK1-transduced MSCs on immune-mediated renal diseases and spontaneous lupus nephritis.

## Materials and Methods

### Bone marrow mesenchymal stem cell isolation and culture

MSCs were isolated as previously described and characterized by flow cytometry [20], [21]. Firstly, Femurs and tibias were aseptically removed from hind limbs by washing with 70% ethanol. BM was flushed from the shaft of the bone with Dulbecco's modified Eagle's medium (DMEM; Sigma-Aldrich, St. Louis, MO) containing 5% fetal bovine serum (Invitrogen, Carlsbad, CA) and penicillin (100 U/ml)-streptomycin (0.1 mg/ml) (Invitrogen, Carlsbad, CA), and then filtered through a 100-µm (pore size) sterile filter (Falcon; BD Biosciences, San Jose, CA) to produce a single cell suspension. MSCs were recovered from BM by their tendency to adhere tightly to plastic culture dishes. Filtered BM cells were plated in cell culture flask containing DMEM plus 10% FBS and penicillin (100 U/ml)-streptomycin (0.1 mg/ml) and cultured in a 5% CO_2_ incubator at 37°C. The cultured cells were replenished with fresh medium every three days. MSCs were identified using six surface markers (CD11b, CD29, CD34, CD44, CD45 and Sca-1) with fluorescence-activated cell sorter (FACS) analysis. CD29, CD44 and Sca-1 were used as positive markers, while CD11b, CD34 and CD45 were used as negative markers.

### MSC cell line with constitutive expression of human kallikrein-1

The coding region of human kallikrein-1 (hKLK1) gene was amplified by reverse transcription polymerase chain reaction (RT-PCR) and cloned into a retroviral vector, pMSCV (Clontech Laboratories Inc, Mountain view, CA). The recombinant pMSCV-hKLK1 was transfected into PT67 packaging cells and the virus was collected after 48 hr of transfection. Cultured MSCs in passage 5 were infected with pMSCV-hKLK1 virus for 48 hr, and then selected with 600 µg/ml of G418 for 2 weeks. Expression of recombinant hKLK1 in MSCs was identified by QPCR and western blot. MSCs infected with pMSCV vector were used as control.

### H_2_O_2_ treatment of hKLK1-MSCs and vector-MSCs

Unmodified MSCs, hKLK1 transduced MSCs (hKLK1-MSCs) and pMSCV vector transduced MSCs (vector-MSCs) were seeded in separate wells of the 6-well plates. When the cells were 60–70% confluent, they were treated with 0.5 mM hydrogen peroxide (H_2_O_2_) for 6 hr. The apoptotic cells were assayed using in situ cell death detection kit (Roche, Indianapolis, IN) following the manufacturer's instructions. The cells were stained with DAPI-containing vectashield mounting medium (Vector laboratories Inc, Burlingame, CA). Apoptotic cells were identified by their distinct condensed nuclei. Images were acquired using Leica DFC420 microscope and Application Suite Advanced Fluorescence software (Leica, Buffalo Grove, IL).

### Animal experiments

All mice were housed at a constant room temperature and humidity and had free access to water and food. All experiments were performed according to the guidelines of the Institutional Animal Care and Use Committee and were approved by the University of Texas Southwestern Medical Center Animal Care and Use Committee in Dallas, TX. No human subjects are studied in this work.

### The anti-GBM induced nephritis mouse model

2-month-old female *129X1/svj* mice (Jackson Lab, Bar Harbor, ME, USA) were used to evaluate the protective effect of hKLK1-MSCs against anti-GBM induced nephritis. 21 mice were randomly divided into 3 groups of 7 mice per group. All mice were subjected to anti-GBM disease as described previously [7]. The mice in Group 1 were injected once with 10^6^ hKLK1-MSCs, Group 2 were injected with the same numbers of vector-MSCs and Group 3 were injected with equal volume of PBS. Blood and urine samples were collected from each mouse at day 0, day 14 and day 21 after anti-GBM disease induction. All mice were sacrificed on day 21 and kidneys were removed and snap frozen in liquid nitrogen or fixed in formaldehyde for immunohistochemical analysis. hKLK1 expression in serum was measured by western blot. Kidneys and other organs were collected on day 21 for the measurement of hKLK1 by QPCR and western blot.

### The lupus nephritis mouse model

*B6.Sle1.Sle3* mice were aged to 8-month-old. 18 female mice were divided into three groups of 6 mice in each group. Group one were injected once with 10^6^ hKLK1-MSCs via tail vein injection, group two were injected with same numbers of vector-MSCs and group three were injected with equal volume of PBS as control. Blood and urine samples were collected on day 0 (before injection) and day 28 to measure proteinuria and BUN. On day 28, all mice were sacrificed and kidneys were excised. One half of each kidney was snap frozen in liquid nitrogen and stored at −80°C and the other half was fixed in formaldehyde for preparation of paraffin-embedded renal sections. hKLK1 expression in blood, kidney and other organs was also measured by QPCR and western blot.

### Measurement of proteinuria and serum BUN

24-hour urine samples were collected from all experimental mice using metabolic cages with free access to water. Proteinuria was determined using Coomassie Plus protein assay kit (Thermal scientific, Rockford, IL). The serum BUN was measured with urea nitrogen kit (Sigma-Aldrich, St. Louis, MO). Measurement was performed according to the manufacturer's instruction.

### Renal pathology

Kidney tissues were fixed in 4% formaldehyde, and dehydrated, and then paraffin embedded. Tissue sections were stained with Periodic Acid Schiff (PAS). Kidney sections were examined in a blinded manner and scored to evaluate the degree of nephritis. The severity of glomerulonephritis (GN) was graded on a 0–4 scale as follows: 0, normal; 1, mild increase in mesangial cellularity and matrix; 2, moderate increase in mesangial cellularity and matrix, with thickening of the GBM; 3, focal endocapillary hypercellularity with obliteration of capillary lumina and a substantial increase in the thickness and irregularity of the GBM; and 4, diffuse endocapillary hypercellularity, segmental necrosis, crescents, and hyalinized end-stage glomeruli. The severity of tubulointerstitial nephritis (TIN) was graded on a 0–4 scale, based on the extent of tubular atrophy, inflammatory infiltrates, and interstitial fibrosis, as detailed previously [22].

### Renal immunohistochemical staining

Immunohistochemical staining was performed on formalin-fixed, paraffin-embedded kidney sections as described previously [23]. The infiltration of intrarenal leukocytes within the glomeruli and interstitial regions were enumerated by staining the kidney sections with antibodies to T-lymphocytes (CD3; 1∶300 dilution, Abcam, San Francisco, CA) and macrophages (Iba1; 1∶800 dilution, Abcam, San Francisco, CA). The standard method was used for immunohistochemical staining following the manufacturer's instructions. Iba1 and CD3 positive cells were counted in 20 high power fields (HPF).

### TUNEL staining

TUNEL staining was performed on cultured MSCs and kidney sections using in situ cell death detection kit (Roche, Indianapolis, IN) following the manufacturer's instructions. The sections were also co-stained with DAPI. Finally, tunel-positive apoptotic cells were counted under blinded conditions in 10 consecutive fields to determine the percentage of apoptotic cells.

### Western blot analysis

Western blot analysis for the cytosolic fraction of MSCs and tissue extracts was performed using rabbit anti-hKLK1 antibody (1∶500 dilution, Protein Tech Group, Inc, Chicago, IL) to detect the expression of hKLK1. Rabbit anti-GAPDH (Santa Cruz, CA) was used for detection of GAPDH as internal control.

### Quantitative real time polymerase chain reaction

Total RNA was extracted from mouse kidney tissue with RNeasy kit (Qiagen, Valencia, CA). RNA was transcribed to obtain cDNA. QPCR were carried out on a 7900HT real-time PCR system (Applied Biosystems) with the universal PCR reaction system under standard thermal cycler conditions. Each sample was run in triplicate. 18S rRNA was used as an internal control. Taqman assay IDs include: CCL2 (Mm00441242_m1), CCL5 (Mm01302428_m1), CCL7 (Mm00443113_m1), CCR5 (Mm01216171_m1), CD40lg (Mm00441911_m1), CXCL12 (Mm00445553_m1), Fas1 (Mm00438864_m1), IL-1β (Mm00434228_m1), IL-6 (Mm99999064_m1), MMP-2 (Mm00439498_m1), NFκB (Mm00476361_m1), TGFβ1 (Mm00436952_m1), TNF (Mm99999068_m1), KLK1 (Hs00158490_m1) and 18S rRNA (Hs03003631_g1).

### Multiplex cytokine assays

Mouse cytokine Magnetic 20-Plex Panel (Life Technology, CA) was used for cytokine detection using Magpix System (Luminex, Austin, TX). The levels of 20 cytokines including FGF-basic, GM-CSF, IFN-γ, IL-1α, IL-1β, IL-2, IL-4, IL-5, IL-6, IL-10, IL-12 (p40/p70), IL-13, IL-17, IP-10, KC, MCP-1, MIG, MIP-1α, TNF-α and VEGF in serum were analyzed using the Mouse cytokine Magnetic 20-plex kit. The assays were performed according to the manufacturer's specifications. Standard curves were generated by using reference concentrations supplied by the manufacturer. Each assay was done in triplicate and a minimum of 3 mice were assayed in each group.

### Statistical analysis

Data were subjected to analysis of variance (ANOVA) and Student's t-test using Prism 5. Group data were expressed as means ± SD. Values of all parameters were considered significantly different at a value of p<0.05.

## Results

### Generation and characterization of stable MSC cell line with constitutive expression of hKLK1

**Figure 1:**
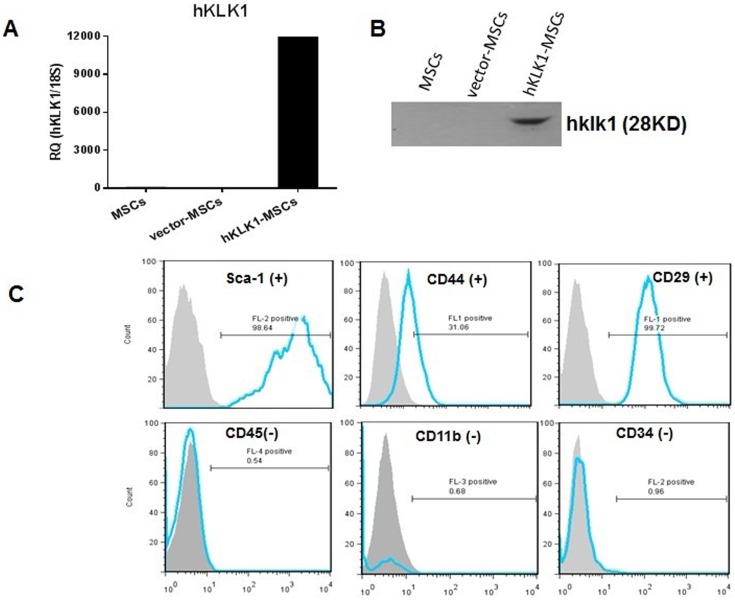
hKLK1-MSCs with constitutive expression of hKLK1. (A, B) Detection of hKLK1 expression in MSCs by QPCR (A) and western blot (B). (C) Immunophenotypic characterization of MSCs. *In vitro* cultured hKLK1-MSCs were stained with antibodies against different cell surface antigens and analyzed by flow cytometry. Gray-filled histograms show flow cytometric analysis of cells stained with isotype control whereas blue thick lines display cells incubated with specific antibodies: Sca-1, CD44, CD29 are positive MSC markers, and CD45, CD11b, CD34 are negative MSC markers.

MSCs isolated from mouse bone marrow were cultured for 5 passages and then transduced with pMSCV-hKLK1. The MSC cells with constitutive expression of hKLK1 were selected by neomycin resistance. The hKLK1-MSC cell line with stable expression of hKLK1 was established and expanded *in vitro*. The expression of hKLK1 in hKLK1-MSCs was confirmed by QPCR and western blot (Fig. 1A and B). After 5 passages, the hKLK1-MSCs still retained their surface properties as characterized by staining with the specific cell surface markers, Sca-1, CD44, CD29, CD34, CD45 and CD11b (Fig. 1C).

### Detection of hKLK1 expression in the kidney of hKLK1-MSC transplanted mice

**Figure 2:**
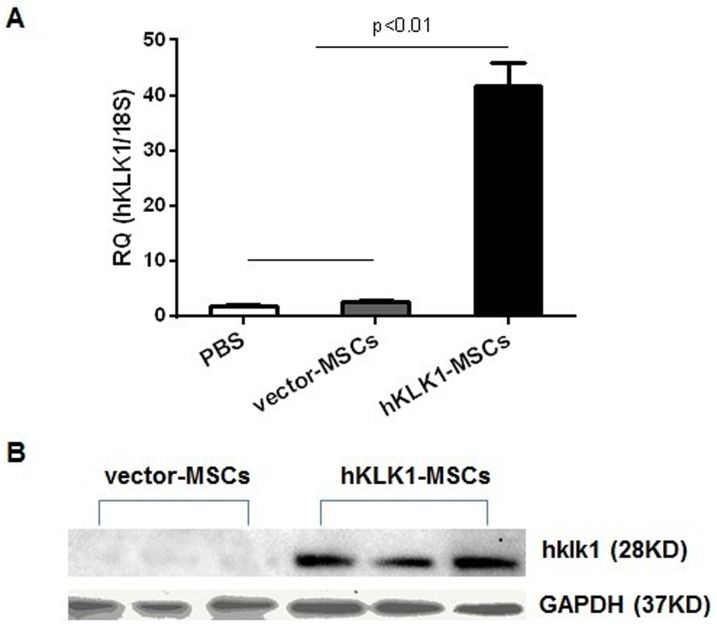
hKLK1 expression in the kidney of *B6.Sle1.Sle3* mice receiving hKLK1-MSCs. (A) hKLK1 mRNA expression was detected by QPCR in the kidney of *B6.Sle1.Sle3* mice injected with hKLK1-MSCs, but not in vector-MSCs or PBS-injected *B6.Sle1.Sle3* mice (*p<0.01*). (B) hKLK1 protein expression was detected by western blot in the kidneys of hKLK1-MSCs injected *B6.Sle1.Sle3* mice, but not in vector-injected *B6.Sle1.Sle3* mice. Three mice were examined for each group.

10^6^ hKLK1-MSCs or vector-MSCs were transplanted into 2-month-old *B6.Sle1.Sle3* mice via tail vein injection and monitored for 3 or 4 weeks. The expression of hKLK1 in kidneys and other tissues was measured by QPCR and western blot at the end of experiment. The hKLK1 expression was detected in the kidneys of hKLK1-MSC injected *B6.Sle1.Sle3* mice but not in vector-MSCs injected mice (Fig. 2A and B). Among the other tissues, hKLK1 expression was only detected in the lung and heart of hKLK1-MSCs recipient mice, but not in spleen, liver, brain and muscle (data not shown).

### hKLK1-MSCs attenuated anti-GBM antibody induced nephritis

**Figure 3:**
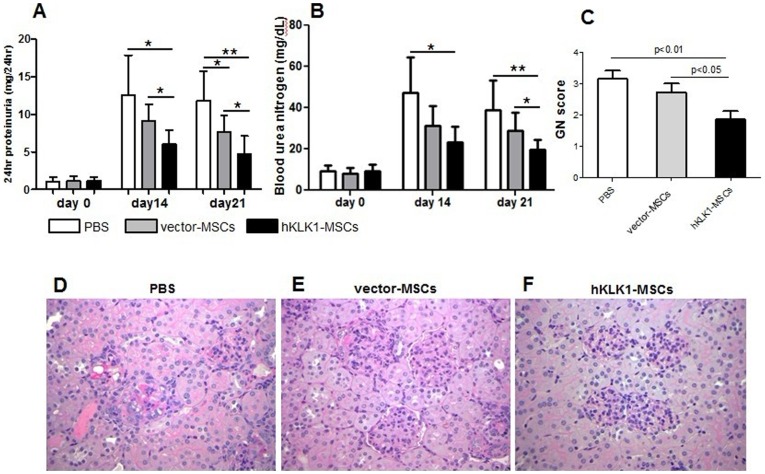
hKLK1-MSCs transfer suppressed anti-GBM induced nephritis in *129/svj* mice. *129/svj* mice were challenged with anti-GBM antibody on day 0 and then treated with hKLK1-MSCs, vector-MSCs or PBS (sham control). Proteinuria (A) and serum BUN (B) were measured on day 0, day 14 and day 21. The values represent the average of 7 mice in each group. *p<0.05, **p<0.01. (C) Renal sections were evaluated for glomerulonephritis (GN) under light microscopy on day 21. (D–F) Shown are periodic acid schiff-stained, formalin-fixed, paraffin-embedded renal sections from *129/svj* mice treated with PBS (D), vector-MSCs (E), or hKLK1-MSCs (F). Images are representative of sections from at least 6 mice in each study group (Original magnification 400×).

The protective effect of hKLK1-MSCs was first evaluated in the model of anti-GBM induced nephritis. Three groups of *129/svj* mice subjected to anti-GBM antibody challenge were injected with 10^6^ hKLK1-MSCs, or equal number of vector-MSCs or PBS as control, and monitored over a period of 21 days for evidence of renal disease. On day 0, prior to anti-GBM challenge, all mice exhibited baseline levels of proteinuria (<2 mg/24 hr). Upon challenge with anti-GBM sera, the level of proteinuria increased in all groups compared with baseline, with the highest level in PBS treated group followed by vector-MSCs and hKLK1-MSCs treated groups (Fig. 3A). On day 14, the PBS group exhibited proteinuria in the range of 12.55±5.31 mg/24 hr, compared with 9.12±2.20 mg/24 hr in vector-MSCs group (*p = 0.17*) and 6.02±1.85 mg/24 hr in the hKLK1-MSCs treated group (*p = 0.02*). On day 21, proteinuria was still maintained at 11.8±3.97 mg/24 hr in the PBS group, but was further reduced to 7.68±2.18 mg/24 hr in the vector-MSCs group (*p = 0.05*) and 4.68±2.43 mg/24 hr in the hKLK1-MSCs group (*p<0.01*). The data indicated that the hKLK1-MSCs treated mice exhibited significantly reduced proteinuria compared with both PBS and vector-MSCs treated mice on day 14 and day 21 (*p<0.05*). The vector-MSCs injected mice also showed reduced level of proteinuria compared with PBS controls on both time points, but did not attain significant level until day 21 (*p = 0.05*) (Fig. 3A).

BUN was also measured in all mice. Upon challenge with anti-GBM antibody, the level of BUN increased in all groups compared to baseline (Fig. 3B). On day 14, the PBS injected mice exhibited an average BUN value of 47.15±7.09 mg/dL, the vector-MSCs injected mice showed decreased levels of BUN (31.25±3.85 mg/dL, *p = 0.08*), whereas the hKLK1-MSCs injected mice exhibited the lowest BUN (23.42±2.98 mg/dL, *p<0.01*). Similar results were also observed in the samples collected on day 21 (Fig. 3B). Statistically, only the hKLK1-MSCs injected mice showed significantly reduced levels of BUN compare with PBS controls (*p<0.05*) on both day 14 and day 21. Between the vector-MSCs and hKLK1-MSCs treated groups, the latter group exhibited significantly lower levels of BUN on day 21 (*p = 0.048*).

Histological analysis was performed to gauge the renal pathology. The mice in PBS-treated control group developed severe proliferative glomerulonephritis (GN score>3) and mild to moderate tubulointerstitial nephritis (Fig. 3C and D). The vector-MSCs treated group exhibited moderate GN (GN score between 2 and 3) with mild tubulointerstitial nephritis (Fig. 3C and E), whereas the mice receiving hKLK1-MSCs exhibited ameliorated GN (score<2) and tubulointerstitial nephritis (Fig. 3C and F). Compared to the PBS and vector-MSCs treated control mice, the hKLK1-MSCs treated mice showed significantly reduced glomerular proliferation, intracapillary hypercellularity and crescent formation, as exemplified in Fig. 3D–F. Collectively, the above functional readouts and histological findings indicated that hKLK1 transduced MSCs have a stronger impact on dampening autoantibody-initiated glomerulonephritis.

### hKLK1-MSCs ameliorated lupus nephritis in B6.Sle1.Sle3 mice

**Figure 4:**
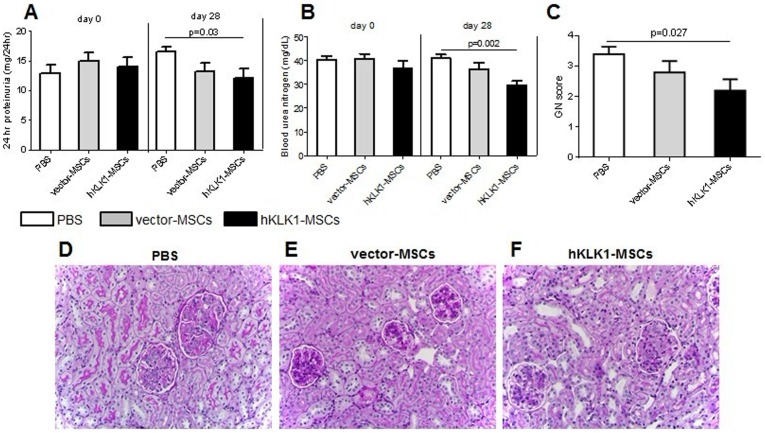
hKLK1-MSCs transfer ameliorated spontaneous lupus nephritis in *B6.Sle1.Sle3* mice. 8-month old female *B6.Sle1.Sle3* mice were treated with 10^6^ vector-MSCs, the same numbers of hKLK1-MSCs, or equal volume of PBS, respectively, via tail vein injection. Proteinuria (A) and serum BUN (B) were measured on day 0 (before treatment) and day 28 (after treatment). Each bar represents the average of 6 mice in each group. (C) Kidneys were collected on day 28 and renal GN score was evaluated under light microscopy. (D–F) Shown are periodic acid schiff-stained, formalin-fixed, paraffin-embedded renal sections from *B6.Sle1.Sle3* mice treated with PBS (D), vector-MSCs (E), or hKLK1-MSCs (F). Images are representative of sections from at least 5 mice in each study group (Original magnification 400×).

Next we tested if hKLK1-MSCs had any therapeutic effect on spontaneous lupus nephritis. The mouse model used for this study was *B6.Sle1.Sle3*, a lupus-prone double congenic strain carrying the Sle1 and Sle3 lupus susceptibility loci which drive autoantibody production and lupus nephritis. The mice were 8-month-old and treated with PBS, vector-MSCs or hKLK1-MSCs, respectively. On day 0 prior to treatment, all three groups of mice showed similar levels of proteinuria with average values of 12.88±3.24, 14.92±3.16 and 14.04±3.43 mg/24 hr in the PBS, vector-MSCs and hKLK1-MSCs treated groups, respectively. 28 days after treatment, proteinuria in the PBS group increased to 16.58±1.62 mg/24 hr, whereas vector-MSCs and hKLK1-MSCs treated groups exhibited 13.26±3.14 mg/24 hr (*p = 0.07*) and 12.1±3.56 mg/24 hr (*p = 0.03*) of proteinuria, respectively (Fig. 4A). Statistically, only hKLK1-MSCs treated mice showed significant reduction in proteinuria compared with PBS controls (*p = 0.03*).

Similarly, the BUN levels on day 0 prior to the treatment were 40.42±3.24, 40.94±4.24 and 36.82±6.67 mg/dL in the PBS, vector-MSCs and hKLK1-MSCs treated groups, respectively. 28 days after treatment, the BUN level in the PBS treated group was maintained at 40.98±3.85 mg/dL level. However, both the vector-MSCs and hKLK1-MSCs treated groups exhibited reduced levels of BUN (36.38±6.32 mg/dL and 29.72±3.80 mg/dL, respectively, Fig. 4B). Again, the hKLK1-MSCs treated mice showed more significant reduction in BUN compared with the PBS control group (*p = 0.002*) and vector-MSCs treated group (*p = 0.08*), indicating that the hKLK1-MSCs had the most potent protective effect.

Renal histological analysis provided further evidences of the protective effect of hKLK1-MSCs on lupus nephritis. As shown in Fig. 4C–F, hKLK1-MSCs treated mice exhibited mild to moderate mesangial hypercellularity with patent or narrowed capillary lumina and slightly thickened capillary walls (Fig. 4F) with a GN score 2.2±0.8 (Fig. 4C), whereas the mice in the PBS control group showed severe renal injury characterized by diffuse global intracapillary hypercellularity with narrowed or obliterated capillary lumina and thickened glomerular capillary walls (Fig. 4D), with a mean GN score of 3.4±0.5 (Fig. 4C). The vector-MSCs treated group also showed a mild renal pathological change with a mean GN score of 2.8±0.8 (Fig. 4E and C).

### hKLK1-MSCs resisted H_2_O_2_ induced apoptosis and renal cell apoptosis in lupus nephritis

**Figure 5:**
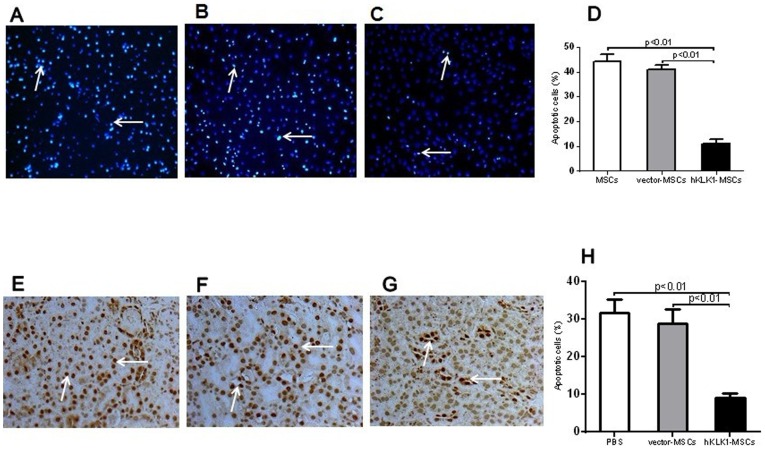
hKLK1-MSCs were resistant to H_2_O_2_ induced apoptosis and hKLK1-MSCs transplantation reduced renal cell apoptosis in *B6.Sle1.Sle3* mice. (A–C) Detection of apoptosis by TUNEL staining of *in vitro* cultured unmodified MSCs (A), vector-transduce MSCs (B) or hKLK1-transduced MSCs (C) treated with 0.5 mM H_2_O_2_ for 6 hr. The apoptotic cells appear as intense fluorescent signals (arrow). (D) Plotted are the percentages of apoptotic cells in each group. (E–G) Detection of apoptotic cells in the kidneys of *B6.Sle1.Sle3* mice treated with PBS (E), vector-MSCs (F) or hKLK1-MSCs (G) for 28 days by TUNEL staining. Apoptotic cells appear as dark brown (arrow). (H) Plotted are the percentages of apoptotic cells in the kidney. Data shown are representative of data from 5 mice in each group.

To test if hKLK1 transduced MSCs were more resistant to oxidative stress, *in vitro* cultured hKLK1-MSCs and vector-MSCs, as well as unmodified MSCs were treated with 0.5 mM H_2_O_2_ for 6 hr and assayed for apoptosis using TUNEL staining. The data indicated that hKLK1-MSCs are significantly less susceptible to H_2_O_2_-induced apoptosis than unmodified MSCs and vector-MSCs (Fig. 5A–D). There was no significant difference between vector-MSCs and unmodified MSCs, suggesting that the resistance to ROS induced apoptosis in MSCs was medicated by over-expression of hKLK1.

The anti-apoptotic effects of hKLK1-MSCs were also observed in mice with lupus nephritis and anti-GBM-induced nephritis. The *B6.Sle1.Sle3* mice injected with hKLK1-MSCs and vector-MSCs were assayed for renal cell apoptosis using TUNEL staining. Compared with PBS injected controls, the vector-MSC injected mice showed similar numbers of apoptotic cells (∼30%) in the renal glomeruli and tubular regions and there was no difference between these two groups. However, *B6.Sle1.Sle3* mice receiving hKLK1-MSCs exhibited fewer apoptotic cells in the kidneys (Fig. 5G) than the mice receiving vector-MSCs or PBS (Fig. 5E and 5F), and these differences were significant (p<0.05, Fig. 5H). Similar results were also noticed in mice with anti-GBM-induced nephritis treated with hKLK1-MSCs or vector-MSCs (data not shown).

### hKLK1-MSCs suppressed inflammatory cell infiltration in anti-GBM induced and lupus nephritis

**Figure 6:**
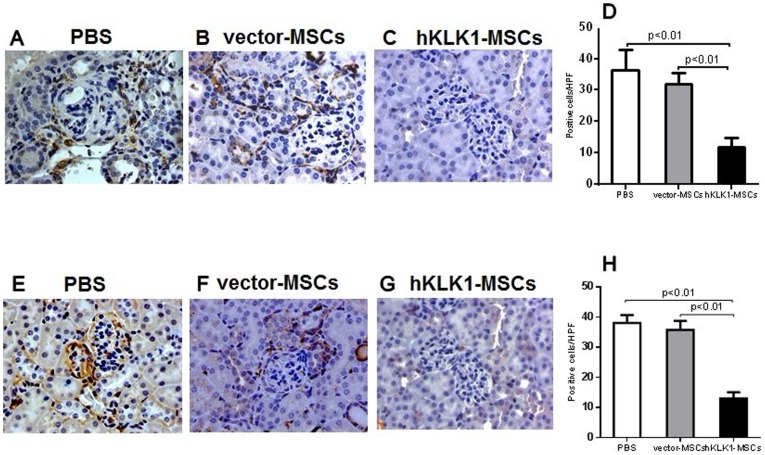
hKLK1-MSC transfer reduced renal macrophage and T-lymphocyte infiltration. Immunohistochemical staining on formalin-fixed, paraffin-embedded kidney tissue sections. Kidney sections from *B6.Sle1.Sle3* mice treated with PBS (A, E), vector-MSCs (B, F) or hKLK1-MSCs (C, G) were stained with macrophage specific anti-Iba1 antibody (1∶800 dilution), or T-lymphocyte specific anti-CD3 antibody (1∶300 dilution). D and H are the percentage of Iba1 positive cells (D) and CD3 positive cells (H). Data shown are representative of 6 mice in each group (Original magnification 400×).

To evaluate if the renal protective effect of hKLK1-MSCs was related to their ability to regulate immune cell infiltration into the kidney, we investigated renal macrophage and T-lymphocyte infiltration in *B6.Sle1.Sle3* mice receiving hKLK1-MSCs, vector-MSCs or PBS using antibodies to Iba1 and CD3. Compared with PBS-treated control mice which showed more robust macrophage (36±4 cells/HPF, Fig. 6A) and T-lymphocyte (38±2 cells/HPF, Fig. 6E) infiltration into the glomeruli and interstitial regions, the vector-MSCs treated mice showed marginally reduced macrophage (32±2 cells/HPF, Fig. 6B) and T-lymphocytes (36±2 cells/HPF, Fig. 6F), and these differences were not significant. However, the hKLK1-MSCs treated mice exhibited significantly reduced macrophages and T-lymphocytes in the glomeruli and renal interstitium (Fig. 6C and 6G) with less Iba1 positive cells (11±3 cells/HPF) and CD3 positive cells (13±1 cells/HPF) (Fig. 6D and 6H, *p<0.01*).

### hKLK1-MSCs transplantation down-regulated the expression of inflammatory chemokines and apoptosis-related cytokines

**Figure 7:**
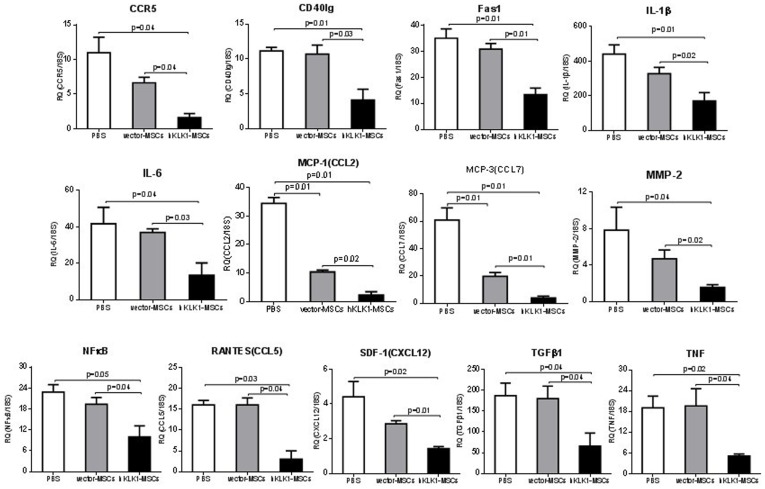
hKLK1-MSCs transfer down-regulated the expression of inflammatory cytokines and apoptosis associated cytokines in mouse kidney. The expression of 13 genes representing proinflammatory cytokines, chemokines and apoptotic factors in the kidneys were measured by QPCR in *B6.Sle1.Sle3* mice treated with PBS, hKLK1-MSCs or vector-MSCs. Total renal RNA was extracted 28 days after treatment. Taqman assay was performed using an ABI 7900HT real-time PCR system. RQ (relative quantity) represents the mean of 5 samples per group. Error bars denote SD.

We next investigated the gene expression of 13 proinflammatory and apoptosis related cytokines in the kidneys of *B6.Sle1.Sle3* mice treated with hKLK1-MSCs, vector-MSCs, or PBS using Taqman real-time PCR assays. As depicted in Fig. 7, hKLK1-MSCs showed the most potent effect, significantly suppressing the expression of all examined genes compared with vector-MSCs and PBS controls (*p<0.05*). The vector-MSCs also showed suppressive effect on most of the cytokines, although only the changes in MCP-1/CCL2 and MCP-3/CCL7 reached significance compared with the PBS controls (p<0.05).

**Figure 8:**
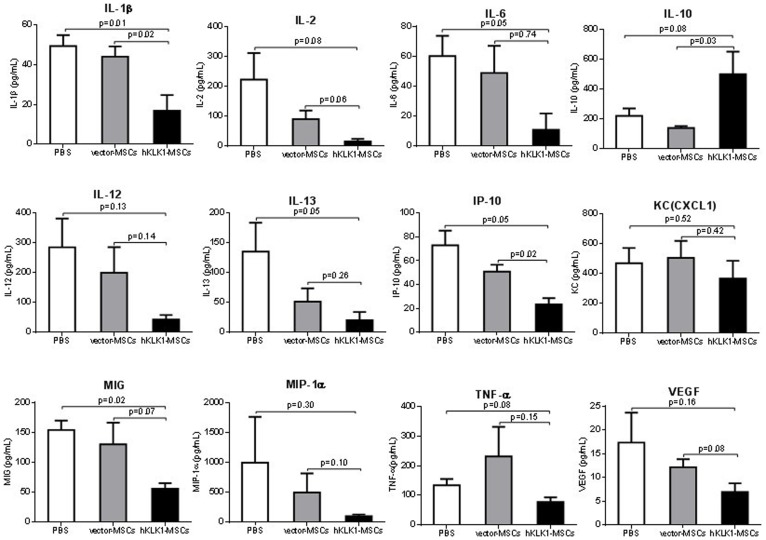
Levels of inflammatory cytokines in mouse serum measured using a Luminex multiplex Assay. 8-month-old *B6.Sle1.Sle3* mice were injected with PBS, 10^6^ hKLK1-MSCs or vector-MSCs and sera were collected on day 28 after treatment. IL-10 expression was increased, while IL-1β, IL-2, IL-6, IL-12, IL-13, IP-10, KC, MIG, MIP-1α, TNF-α and VEGF were decreased in the sera of hKLK1-MSCs treated mice compared with both PBS treated mice and vector-MSCs treated mice. Each bar represents the mean of 3 mice. Error bars denote SD.

A luminex-based multiplex assay was further used to detect the cytokine expression at the protein level. Again, hKLK1-MSCs showed a higher level of suppression of a large number of cytokines including IL-1β, IL-2, IL-6, IL-12, IP-10, MIG, MIP-1α, KC and TNF-α, compared with vector-MSCs, with the exception of IL-10 which was increased in hKLK1-MSCs treated mice (Fig. 8). Although vector-MSCs also showed suppressive effect on some of the cytokines, there was no statistical significance compared with PBS controls.

## Discussion

The Kallikrein-kinin system (KKS) constitutes a multigene family of serine esterases with a wide spectrum of biological functions, including vasodilation, NO production, and cAMP activation [24]–[26]. It's been reported that KKS plays a protective role against cerebral and renal injury caused by ischemic stroke [27], [28], diabetic nephropathy [29]–[31], salt-induced hypertensive glomerulosclerosis and gentamicin-induced nephrotoxicity [32]–[34]. Our previous studies have also demonstrated that kallikreins acting through the kinin B2 receptor exhibited therapeutic effect against anti-GBM induced nephritis [7]. In this study, we further confirmed that the delivery of hKLK1 into the kidneys by genetically modified-MSCs attenuated spontaneous lupus nephritis in mice. Taken together, these data suggest that klks have the potential to be used as therapeutic agents in a wide variety of kidney diseases. Exploring the approaches for efficiently delivering klks into the target organs and identifying the potential molecular pathways mediated by klks are the main goals of this study.

MSC transplantation has been reported to be effective for countering ischemic kidney injury [15] and for ameliorating mesangioproliferative glomerulonephritis in mice [35]. Several studies have shown that bone marrow derived MSCs have protective effect against SLE in both mouse models and in human diseases [36], [37]. In this study, we compared the effect of vector transfected MSCs (vector-MSCs) with hKLK1 transfected MSCs (hKLK1-MSCs) on anti-GBM induced nephritis and lupus nephritis. Our data demonstrated that hKLK1-MSCs have significantly enhanced protective effect against both anti-GBM induced and lupus nephritis compared with vector-MSCs. We showed that hKLK1-MSCs strongly suppressed the renal cell apoptosis and inflammatory cell infiltration into the kidneys of nephritic mice. These findings are consistent with the results by Hagiwara, *et al*, who showed that tissue kallikrein-modified MSCs transfer provided enhanced protection against acute ischemic kidney injury [19]. It should be pointed out that the vector-alone transfected MSCs also showed potent protective effect against lupus nephritis, although not as marked as hKLK1-transfected MSCs. Other researchers have reported that MSCs could possess immunomodulatory effects on T and B lymphocytes, natural killer and antigen-presenting cells [38]–[40]. Experimental data has indicated that MSC transplantation may suppress the excessive activation of B cells via inhibiting BAFF [41]. MSCs could also inhibit T cell activation by suppressing Akt/GSK3β signaling [42], or by shifting the Th1/Th2 balance. The immunosuppressive effect of MSCs could involve both cell contact and secretion of soluble factors, such as indoleamine 2, 3-dioxygenase, prostaglandin E2, nitric oxide, transforming growth factor (TGF)-β1, IL-10, soluble HLA-G, and IL-1 receptor antagonists [35], [42]–[45]. In human SLE, the transplantation of either allogeneic or autologous MSCs derived from bone marrow or umbilical cord also increases Treg cells, suggesting that this may be one of the mechanisms through which MSC-mediate disease improvement [37].

We have observed that the GFP-labeled MSCs migrated to the tubular region and proliferated locally within the kidneys of *B6.Sle1.Sle3* mice with lupus nephritis but not in B6 mice (data not shown), indicating that MSCs have the propensity to migrate to the site of tissue injury and potentially ameliorate injury by producing renotrophic factors [46]–[48]. Engineered MSCs with hKLK1 expression provided enhanced protection, suggesting that apart from the innate protective effect of MSCs, the engineered MSCs can be used as vehicles to deliver therapeutic agents such as hKLK1 to injured end-organs. However, given that the trafficking efficiency of MSCs to inflamed organs is only modest, strategies that could enhance migration are clearly warranted.

The molecular mechanisms that underlie the protective effect of KLK1 transduced MSCs have not been fully explored. There are indications from the literature that the beneficial effects of kallikrein-kinins may be mediated through the inhibition of oxidative stress, apoptosis, inflammation and fibrotic responses [26], [32]–[34], [49]. It has also been reported that kallikreins and kinins suppressed H_2_O_2_-induced apoptosis and increased cell viability and Akt phosphorylation in cultured renal tubular cells [19]. Similarly, we also observed that klk transduced MSCs are more resistant to H_2_O_2_ induced apoptosis *in vitro*. Moreover, we also find that hKLK1-MSCs down regulated apoptosis-associated genes, such as CD40lg, Fas1 and TNF, and significantly suppressed renal tubular cell apoptosis. Given that oxidative stress induced apoptosis in glomerular cells may correlate with immunoserological and histopathological activity in lupus [50]–[52], this is certainly another mechanism through which klk may have operated in our model.

The inhibition of oxidative stress in lupus nephritis after hKLK1-MSCs implantation may also contribute to a subsequent reduction in inflammatory cell infiltration and kidney injury. It's been reported that anti-apoptotic agents can not only block the early onset of apoptosis, but also reduce inflammation and tissue injury [52]. One of the characteristic features in lupus nephritis is glomerular inflammation caused by infiltration of macrophages [53]. In the course of lupus nephritis, various proinflammatory mediators may influence tissue inflammation [3]. Elevated expression of various chemokines has been identified in experimental autoimmune nephritis in mice and spontaneous lupus nephritis [54]–[57]. These chemokines can serve as chemical mediators and recruit various leukocyte subsets in the affected kidneys. Our study demonstrated that the transfer of hKLK1-MSCs into mice with nephritis, reduced the numbers of renal-infiltrating macrophages and T-lymphocytes, accompanied by the down-regulation of a series of proinflammatory cytokines/chemokines, including RNATES, SDF-1, MCP-1, MCP-3, CCR5, MMP-2, IL-1β, IL-6, TGFβ1 and NFκB. We have shown previously that RANTES was essential for immune-mediated glomerulonephritis [58], and we believe the down-regulation of this cytokine is one contributing factor to the observed disease improvement. On the other hand, IL-1 has been reported to play an important role in inflammatory glomerular disease [59], [60]. IL-1 is elevated in experimental glomerular disease and administration of IL-1 accentuates the renal injury, associated with directly stimulating the proliferation of mesangial cells, as well as indirectly exacerbating injury by increasing the production of other inflammatory mediators such as IL-6, TNF-α and ICAM [59]–[61]. Another down-regulated molecule, MMP-2, belongs to the family of matrix metalloproteinases (MMPs) which are intimately involved in the turnover of major glomerular basement membrane constituents (collagen IV and laminins) [62], [63]. Alterations in the expression and activity of MMPs have been described in a number of renal diseases, suggesting their relevance to the pathogenesis of various glomerulopathies. Regarding the connections between the KKS and proinflammatory mediators, it has been reported that activation of kinin B2 receptor leads to the down-regulation of MCP-1 and VCAM-1, thus inhibiting inflammatory responses in the infarcted heart [49]. Interestingly, the expression of IL-10 was up-regulated in hKLK1-MSCs treated mice compared with vector-MSCs treated mice. IL-10 has been reported to play immune-regulatory roles and its overexpression delays autoantibody production and clinical nephritis in lupus mice [64]. Thus, klk appears to dampen several pathogenic mediators in lupus mice, while augmenting the levels of protective cytokines.

Collectively, the protective effect of hKLK1-MSC may have arisen from the combined beneficial effects of klk and MSCs. Given that MSCs have the propensity to migrate to inflamed tissues, we believe klk-MSCs represent ideal vehicles to ferry renoprotective kallikreins to the inflamed kidneys, without incurring systemic side-effects. Importantly, klk-MSCs appear to counter multiple pathogenic events associated with tissue damage in lupus nephritis, including oxidative stress, inflammatory cell infiltration, and apoptosis. Moreover, this strategy also makes it possible to deliver multiple therapy genes into inflamed target organs, and this has far-reaching implications for the treatment of multiple end-organ diseases.
